# Within-person changes in the aging white matter microstructure and their modifiers: A meta-analysis and systematic review of longitudinal diffusion tensor imaging studies

**DOI:** 10.1162/imag_a_00045

**Published:** 2023-12-14

**Authors:** Andrea Mendez Colmenares, Ben Prytherch, Michael L. Thomas, Agnieszka Z. Burzynska

**Affiliations:** Department of Human Development and Family Studies/Molecular, Cellular and Integrative Neurosciences, Colorado State University, Fort Collins, CO, United States; Department of Statistics, Colorado State University, Fort Collins, CO, United States; Department of Psychology/Molecular, Cellular and Integrative Neurosciences, Colorado State University, Fort Collins, CO, United States

**Keywords:** white matter, longitudinal, aging, healthy aging, MRI, brain, DWI, DTI, diffusion tensor, diffusion imaging, diffusion-weighted imaging

## Abstract

This meta-analysis and systematic review synthesized data from 30 longitudinal diffusion tensor imaging (DTI) studies on the magnitude, direction, spatial patterns, and modifiers of naturally occurring within-person changes in healthy adult white matter (WM) microstructure. Results revealed: (1) significant within-person declines in fractional anisotropy (FA) in the whole WM (*d* = -0.12), genu (*d* = -0.16), and splenium (*d* = -0.13); (2) greater declines in FA associated with older age, longer follow-up times, and female sex; (3) a possible yet inconclusive vulnerability of late-myelinating WM (the “development-to-degeneration” gradient); and (4) factors decelerating (e.g., physical activity and social activities) and accelerating (e.g., vascular risk factors, biomarkers for Alzheimer’s disease, and alcohol consumption) age-related FA changes. Our findings encourage the consideration of WM as a new target for treatments and interventions against cognitive decline and lay the foundation for studying the plastic and regenerative potential of adult WM in clinical trials. Individual differences in WM changes could aid in the preclinical diagnosis of dementia, opening a window for earlier, more effective treatments.

## 1 Introduction

Human white matter (WM) contains mostly myelinated axons, whose properties determine the speed and synchrony in the brain’s transduction and transmission of neural signals (Chorghay et al., 2018). The WM is supported by a complex network of glial cells—oligodendrocytes, astrocytes, and microglia—each cell type plays a distinct role, from myelination and energy supply to immune responses and neuron-glia interactions. This intricate composition, however, makes WM highly susceptible to ischemic injuries due to its limited blood flow compared to gray matter. Specifically, the WM of the brain is particularly sensitive to metabolic, inflammatory, and vascular dysfunction (Levit et al., 2020; Mendelow, 2015), all hallmarks of brain aging, Alzheimer’s disease, and related dementias, and even neuropsychiatric disorders such as schizophrenia (Zhao et al., 2022). This vulnerability of WM is accentuated by the metabolically demanding processes of myelin maintenance and long-distance axonal transport (Bartzokis, 2004; Nave, 2010), which are necessary for efficient action potential conduction and metabolic support of myelinated axons (Morrison et al., 2013). Postmortem studies in healthy older adults have shown that aging is associated with demyelination and decreases in axonal density or diameter (Marner et al., 2003; Mason et al., 2001; Peters, 2002; Tse & Herrup, 2017). Similarly, failed myelin repair (Bartzokis, 2004, 2011) and defects in axonal structure and transportation (Stokin et al., 2005) have been observed in the early stages of Alzheimer’s disease, suggesting that gray matter pathology may be triggered or preceded by WM pathology. Specifically, the “myelin” hypothesis of Alzheimer’s Disease posits that proteinaceous deposits such as amyloid-β aggregates and tau tangles are the by-products of homeostatic myelin repair processes and disruptions to axonal transport (Bartzokis, 2011). Together, alterations in WM microstructure in both healthy aging and neurodegenerative processes result in a structural “disconnection” of distributed neural networks, considered one of the primary mechanisms underlying cognitive decline in healthy aging, Alzheimer’s disease, and related dementias (Bartzokis, 2004; Nasrabady et al., 2018).

However, postmortem histopathological examinations provide no insights into how these changes in WM occur over time and to what extent the magnitude or patterns of within-person progression differs between healthy and pathological aging. These answers can be studied only *in vivo* using non-invasive techniques, such as Magnetic Resonance Imaging (MRI). Therefore, this article aims to synthesize the evidence from longitudinal diffusion tensor imaging (DTI) studies on the magnitude, direction, spatial patterns, and possible modifiers of naturally occurring within-person changes in adult WM microstructure. Specifically, we aimed to address the following questions: What is the magnitude and direction of within-person changes in adult WM microstructure? What are the time periods over which WM microstructural decline can be detected in healthy adults? Do within-person changes in white matter microstructure accelerate with age? Is there regional variability in WM changes? What factors modify within-person changes in the WM?

To date, WM microstructure in aging, Alzheimer’s disease, and related dementias has been studied almost solely using diffusion MRI and predominantly using the DTI model (Harrison et al., 2020; Madden et al., 2012). DTI provides a voxel-wise estimation of the magnitude and directionality of water diffusion. Fractional anisotropy (FA) measures the directional dependence of diffusion, reflecting fiber-orientational coherence within a voxel. Radial diffusivity (RD) and axial diffusivity (AD) represent diffusivity perpendicular and parallel to the main fiber direction, respectively. Finally, mean diffusivity (MD) reflects the overall magnitude of total water diffusion within a voxel (Beaulieu, 2002). The magnitude of diffusion is determined by microstructural elements that may hinder diffusion in any direction, such as density, permeability, and integrity of axonal and myelin membranes, activation of glia, microvasculature, and enlargement or tortuosity of extracellular spaces (Jones et al., 2013). This review focuses on DTI—the most widely used diffusion MRI technique—yet we acknowledge that several more advanced diffusion acquisition and modeling methods have been applied in recent cross-sectional studies.

The study’s first aim was to determine the magnitude and direction of within-person changes in DTI parameters in the adult WM microstructure in older age. Age-comparative (cross-sectional) studies on aging consistently report decreased FA, increased MD, RD, and bidirectional age differences in AD (Burzynska et al., 2010). These age differences have been attributed to loss of “WM integrity,” including loss of myelin and axons (Madden et al., 2012). Furthermore, cross-sectional studies have suggested nonlinear trajectories in diffusion parameters across the lifespan, suggesting protracted development or myelination until middle adulthood. Specifically, FA has been shown to peak between 20 and 42 years of age, followed by a decline, whereas MD shows a minimum at 18–41 years, followed by a steady increase from middle adulthood onwards (Lebel et al., 2012). An analysis of different diffusion parameters in 3,513 generally healthy people aged 45–77 years from the UK Biobank revealed predominantly nonlinear associations with age (Cox et al., 2016). Specifically, an increase in MD and a decrease in FA accelerated typically after age 60 (Cox et al., 2016). Therefore, our central hypothesis was that within-person changes in middle and older age would predominantly involve declines in FA and increases in MD and RD. In addition, we expected these changes to accelerate after the age of 60.

Our second aim was to test the spatial gradients of WM aging. Cross-sectional findings revealed that WM tracts differ in their susceptibility to aging. As a result, several spatiotemporal gradients have been proposed to explain this selective vulnerability. The overarching model, called development-to-degeneration, retrogenesis, or last-in-first-out hypothesis, posits that WM regions that myelinate later in development deteriorate earlier with age, possibly due to greater metabolic demands on late-differentiating oligodendrocytes (Bartzokis, 2004; Bartzokis et al., 2004). Cross-sectional DTI data have lent substantial support for the retrogenesis hypothesis (Brickman et al., 2012), as reflected by studies showing steeper age decline in prefrontal regions and association fibers than in projection fibers (Barrick et al., 2010; Burzynska et al., 2010) and steeper age decline in the most anterior sections of the corpus callosum (Bartzokis, 2004; Head et al., 2004; Salat et al., 2005; Sullivan et al., 2010). Therefore, we hypothesized that late-myelinating WM regions, such as the genu of the corpus callosum, will show the greatest magnitude of within-person declines in FA and increases in RD, possibly reflecting demyelination. In contrast, regions myelinating earlier, such as the corticospinal tract or posterior sections of the corpus callosum, may show FA declines only in later life (i.e., after age 70).

Our third aim was to explore the role of various modifiers of within-person changes in adult WM. We expected chronological age to be the main moderator of declines in WM integrity, with older age correlating with a greater magnitude of decline. Furthermore, given the role of sex hormones in promoting myelination, oligodendrocyte proliferation (Ghoumari et al., 2020; Jure et al., 2019; Mendell & MacLusky, 2018), and modulating brain inflammation (Yilmaz et al., 2019), we believe there could be sex differences in age-related declines in WM. So far, cross-sectional DTI studies have reported greater FA in men (Kochunov et al., 2012; Lebel et al., 2012; Ritchie et al., 2018) or no sex differences across the adult lifespan (Kennedy & Raz, 2009). Thus, our analyses concerning sex differences were exploratory. Other candidate modifiers of WM aging include hypertension (van Dijk et al., 2004), habitual physical activity (Burzynska et al., 2014; Sexton et al., 2016), or APOE genotype (Sudre et al., 2017). In addition, since people with mild cognitive impairment, subjective cognitive impairment and risk of Alzheimer’s disease show higher MD and lower FA compared to healthy older adults (Brueggen et al., 2019), we will also discuss evidence of within-person change in these groups.

Studying within-person changes in adult WM is important given that for decades, WM has been thought to play a passive role in brain function by merely relaying electrical signals between gray matter regions, where information processing occurs. In addition, the adult WM has been considered “static” after reaching maturity, namely, not capable of or involved in neuroplasticity and only prone to deterioration due to age or disease. Recently, rodent studies have shown that cognitive, and motor learning in adult animals requires myelin plasticity (Gibson et al., 2014; Hines et al., 2015; Jeffries et al., 2016; McKenzie et al., 2014; Sampaio-Baptista et al., 2013). However, because the evidence of training-induced changes in adult human WM microstructure is scarce and inconsistent, WM remains rarely considered the primary target for treatments and interventions against cognitive decline (Mendez Colmenares et al., 2021; Sampaio-Baptista & Johansen-Berg, 2017), which is a missed opportunity. We argue that understanding the naturally occurring within-person changes in WM in older age will lay the foundation for studying adult WM’s plastic and regenerative potential in future clinical trials. In this literature review, we also reviewed evidence from clinical studies to assess the malleability of adult WM microstructure with experience, to identify the most promising interventions for inducing change.

Taken together, our overarching hypothesis was that WM microstructure undergoes significant within-person changes during adulthood and aging, and that these changes can be captured noninvasively with DTI. We hypothesized that within-person changes in WM microstructure in older age: (a) involve predominantly declines in FA and increases in MD and RD; (b) are characterized by declines in FA and concurrent increases in MD and RD; (c) follow the development-to-degeneration spatiotemporal pattern, with greater magnitudes of change in late-myelinating regions; (d) are moderated by duration or time until follow-up, sex, hypertension, lifestyle factors, and genetic risk factors for Alzheimer’s disease, and are more pronounced in individuals with mild cognitive impairment or risk of Alzheimer’s disease. To answer these questions, we conducted a comprehensive qualitative review of longitudinal DTI studies and performed a meta-analysis on a subsample of studies that provided sufficient data.

## 2 Methods

Our study was pre-registered in the PROSPERO database as PROSPERO 2021 CRD42021273127.

### 2.1 Search strategy

A systematic search was performed in electronic databases Web of Science and Pubmed up to July 13, 2021. The main search strategy was based on three key components: longitudinal studies, white matter, diffusion tensor MRI, and healthy adult samples. The PubMed database was searched for the terms in either the title or abstract, whereas the Web of Science database was searched for the terms in “topic,” which includes title, abstract, and keywords. We searched for studies in peer-reviewed journals, applying no limitations on publication year or language. Given that researchers use different terms to refer to DTI and may not use the DTI or MRI abbreviations in the abstract or title, we used the broad term “diffusion” in our search query. The PubMed query ("white matter"[Title/Abstract] AND "longitudinal"[Title/Abstract] AND "diffusion"[Title/Abstract] AND "adults"[Title/Abstract]) resulted in 283 hits. The Web of Science query ("white matter"(Topic) and longitudinal (Topic) and diffusion (Topic) AND "adults"(Topic)) resulted in 531 hits. After inspection of the results, we noticed that many hits for “longitudinal” were associated with the longitudinal fasciculus. Therefore, we added the NOT “longitudinal fasciculus” term to both queries, resulting in 126 hits in PubMed and 248 hits for the Web of Science. In addition, reference lists of included studies and relevant reviews were manually searched for additional eligible studies.

### 2.2 Study selection

A.M.C. and A.Z.B. independently screened the title, abstracts, and, where appropriate, full text of identified citations and any disagreements were resolved by consensus. For studies to be included in the systematic review, the following criteria had to be met:

1. Reported DTI parameters (FA, MD, RD, AD) from WM regions collected on at least two occasions per participant. Studies assessing change in only macroscopic measures of WM health (e.g., WM volume or hyperintensity burden) were not included. Both observational longitudinal and clinical trials were considered, but only if they included younger adults AND middle-aged or older adults (i.e., clinical trials in only student/young adult populations (e.g., age 18-25) were excluded). Studies evaluating solely intra- or inter-scanner stability were also excluded. Studies not reporting DTI metrics (i.e., studies reporting only structural connectivity measures) were excluded.
2. Published as an original empirical peer-reviewed journal article. While this may raise susceptibility to publication bias, restricting the search to published results serves as a way to encourage high quality in the included reports. Meta-analyses or review articles on related topics were excluded.
3. Included adult samples of age 18+. Studies including only children and adolescents were excluded.
4. Included cognitively and neurologically healthy adults. Healthy adults generally excluded participants on anxiolytics, antidepressants, or antiepileptics and those consuming over three alcoholic beverages daily. Some studies reported including participants with treated hypertension (Bender, Völkle, et al., 2016; Williams et al., 2019), but this information was not reported in all studies. Animal and patient populations (e.g., schizophrenia, autism, stroke, concussion, substance abuse, pre-hypertension) were excluded, except for studies involving people with mild cognitive impairment, Alzheimer’s disease, and related dementias in older age groups, which were included in the qualitative review.
5. We excluded studies that did not report change (or effect of time) in DTI parameters as a study outcome. These studies included (Fissler et al., 2017; Fletcher et al., 2013; Lampit et al., 2015; Racine et al., 2019), who reported only differences in change between clinical and healthy populations, or (Maltais et al., 2020; Raffin et al., 2021; Scott et al., 2017; Staffaroni et al., 2019) who used change in DTI only as a correlate of change in cognition, brain perfusion, or baseline physical activity. However, we listed these studies in Appendix A and mentioned them in the qualitative review of modifiers of WM change.
6. In addition, we excluded two studies with short follow-up times (<4 weeks) (Chen et al., 2020; Nilsson et al., 2021)

### 2.3 Data selection

**Fig. 1.**
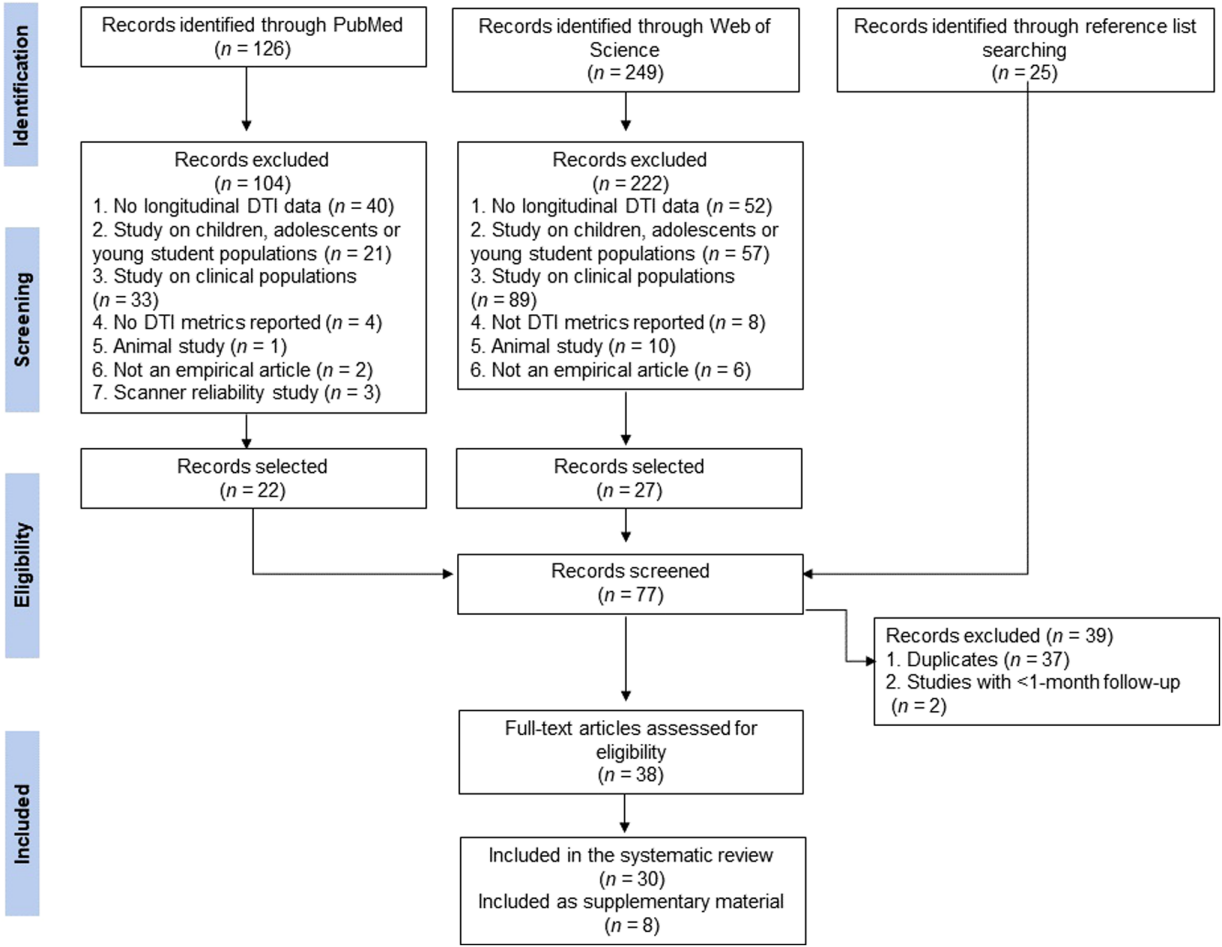
Flow chart of selected studies.

The PRISMA flowchart provides an overview of the number of articles screened, included, and excluded (Fig. 1). We included a total of 30 studies in the systematic review, of which half had sufficient data to be included in the meta-analysis. Missing outcomes were requested by contacting the corresponding authors. We contacted 25 authors with insufficient data in the original publication to calculate standardized mean differences or standard errors and received 13 responses.

Given the variability in reporting all four DTI parameters, we focused only on FA to maximize the number of studies for the meta-analyses. At the same time, other DTI metrics are discussed in the qualitative review.

**Fig. 2.**
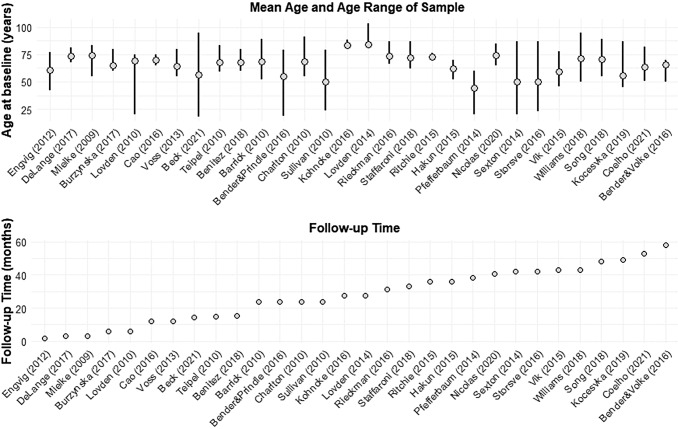
Study characteristics: age range, mean age, and follow-up time.

From the 30 studies included in the review, the median year of publication was 2015 (range 2009–2021). The median sample size was 56, varying from 11 to 2,125. The average baseline age was 65.3 years (range 18–103 years). The mean follow-up time was 27.7 months (range 2-58 months) (Fig. 2).

Studies with overlapping samples were excluded when the same aspect of WM structure was examined in both papers (Kocevska, Cremers, et al., 2019; Kocevska, Tiemeier, et al., 2019). In this case, the study with the largest sample size was first given preference. One study reported multiple follow-up visits (Bender, Völkle, et al., 2016). In this case, for the meta-analysis, we used data from the longest follow-up time. We included six randomized controlled trials with longitudinal DTI data and collected information from the healthy control groups (Burzynska et al., 2017; Cao et al., 2016; de Lange et al., 2017; Engvig et al., 2012; Lövdén et al., 2010; Voss et al., 2013). We excluded one randomized controlled trial without a control group (Clark et al., 2019).

### 2.4 Risk of bias (quality) assessment

A.M.C. and an external reviewer assessed the risk of bias with the NIH quality assessment tool for observational cohort studies, case control studies, and pre-post studies with no control group (*Study Quality Assessment Tools | NHLBI, NIH*, 2013). Studies needed to have clearly defined aims, a clearly specified study population, appropriate inclusion criteria description, ethical approval, and healthy adults recruited from the community (see Appendix B and Appendix C for more details). In addition, A.Z.B. and A.M.C. performed the quality check of the reported MRI methodology and statistics.

### 2.5 Data extraction

A.Z.B. and A.M.C. independently extracted the following details using a structured data abstraction form: MRI method of WM microstructure quantification, study design (number and time between within-person measurements, longitudinal observational vs. intervention), anatomical specificity (global or regional measures of WM microstructure), participant demographics (sample size, age range, age at baseline, percentage of female participants), and results (statistically significant findings, measures of change, and their standard errors, Table 1).

**Table 1.** Characteristics of the qualifying DTI longitudinal observational studies (n = 30).

Authors	Year	Country/study	Follow-up	N, % female	Age (y)	DTI measure	WM regions	Statistics reported	Main results
Mielke et al.	2009	USA	3 m	25 HC, 56%24 MCI, 28%21 mild Alzheimer’s, 28%	M = 74	FA	FX, CING, SCC, CP	M±SE at t1 and t2	FA↓ in CING in MCI; No ∆ in HC
					M = 75				
					M = 76				
Sullivan et al.	2010	USA	2 y	16 HC, 50%	24–4065–79	FA, RD, AD,	6 subsections of CC (tractography), midsagittal & distal sections	M±SD at t1 and t2 (as plots only)	No ∆
Barrick et al.	2010	UK/GENIE study	2 y	73 HC, 41%	50–90, M = 68.3	FA, RD, AD	Whole-skeleton voxelwise analysis; On WM skeleton: CC, IC, EX, CING, SCR	M±SD at t1 and t2, t2-t1	FA↓, RD↑, AD↑
Charlton et al.	2010	UK/GENIE study	2 y	73 HC, 43%	55-91, M = 68.3	FA, MD	Whole WM	Normalized peak height freq., median±SD at t1 and t2	Histograms: FA↓, MD↑ median & kurtosis
Teipel et al.	2010	Germany	13-16 m	11 HC, 36%	60-88 M = 67	FA	Regions on WM skeleton: CC, FX, CING, SLF	Annual %∆	FA↓ in both groups, no time-by-group interaction
				14 MCI, 43%	59-83				
					M = 67.4				
Lövdén et al.	2010	Sweden/COGITO	6 m	HC: 10, 40%	20-30	FA, RD, AD, MD	5 subsegments of CC	M±SD at t1 and t2	No ∆ in HC
			(RCT, cog. training)	HC: 13, 31%	65-76				
				HC RCT: 20, 55%	22-30				
				HC RCT: 12, 58%	65-75				
Engvig et al.	2012	Norway	8 weeks (RCT, memory training)	HC: 20, 55%	42-77	FA, MD	Voxelwise on WM skeleton	% Voxels showing significant ∆, M±SD t2-t1 for significant voxels	MD↑No ∆ in FA
				HC RCT: 21, 52%	M = 60.3				
Voss et al.	2013	USA	1 y (RCT, walking)	HC Control: 35, 60%	60-80	FA, RD, AD	4 lobes on WM skeleton,	M±SD at t1 and t2, Annual %∆	RD↑ in temporal lobe, FA↑ in the frontal lobe. No time x group interaction.
				HC RCT: 70, 64%	M = 65		Voxelwise on skeleton (t2-t1)/t1		
Pfefferbaum et al.	2014	USA	1-8 y (M = 3 y), 2-5t	56 HC, 57%, 46 alcoholics, 40%	20-60, M = 44	FA, RD	Whole-skeleton analysis, post-hoc in clusters representing 27 regions	T, df and p value in clusters showing age differences (M at t1 and t2 only in plots)	FA↓, RD↑ in both groups
Lövdén et al.	2014	Sweden/Swedish national Study on Aging and Care in Kungsholmen (SNAC-K)	2.3 y	HC 40, 55%	81-103, M = 84	FA, MD	6 regions: CING gyrus, CST, Fmaj, Fmin, IFOF, SLF	M±SD at t1 and t2	FA↓, MD↑
Sexton et al.	2014	Norway/Cognition and Plasticity through the Lifespan	3-5 y (M = 3.6 y)	HC 203, 59%	20-87, M = 50.2	FA, MD, RD, AD	Whole-skeleton analysis, 4 lobes, significant clusters	Annual difference maps ((t2-t1)/y follow up), mean % ∆ ((t2-t1)/(t1+t2/2)) ±SD	FA↓, MD↑, RD↑, AD↑
Hakun et al.	2015	USA	3 y	HC 18, 50%	52-70, M = 62.4	FA	Voxelwise in whole WM, Tracts: BCC, GCC	No values reported for voxel-wise paired t-test. Individual %∆ in FA in BCC	FA↓
Ritchie et al.	2015	UK/Lothian Birth Cohort 1936	3 y	HC 488, 47%	72-76, M = 73	FA	12 tracts: GCC, SCC, CING, CING gyri, ARC, UN, ILF, ATR	Factor loadings from latent change model (β and SE), controlled for age and sex	FA↓
Vik et al.	2015	Norway	M = 3.6 y	HC 76, 68%	46-78, M = 59	FA	Tractography: 19 frontal-subcortical, anterior callosal tracts, CST	M±SD of tracts at t1 and t2, annual %∆, parametrized tract M and %∆	FA↓
Bender, Prindle, et al.	2016	USA	2 y	HC 96, 69%, 76 normotensives	19-79, M = 55	FA, RD, AD	13 regions on WM skeleton: GCC, BCC, SCC, dorsal and ventral CING, UN, ALIC, PLIC, SLF, ILF, IFOF, FMaj, Fmin	Latent mean ∆ (β /SE, d, ∆Var: latent variance in ∆ parameter as β /SE) (for all and n = 76 normotensive only)	FA↓↑, RD↓↑, AD↓↑
Bender, Völkle, et al.	2016	USA	1-7 y 0-4 t	HC 35, 55%	50-70, M = 65.4	FA, RD, AD	12 regions on WM skeleton: CING, IFOF, ILF, SLF, UN, ALIC, PLIC, GCC, BCC, SCC, FMaj, Fmin	LME model: β, SD, SE for regions grouped as association, commissural, projection	FA↓, RD↑, AD↑
Storsve et al.	2016	Norway/ Cognition and Plasticity through the Lifespan	3-5 y (M = 3.6 y)	HC 201, 59%	23-87, M = 50	FA, MD, RD, AD	18 major tracts: Fmin, Fmaj, ATR, Angular and cingular CING, CST, ILF, SLF, UN	Annual %∆±SD	AD↓ and FA↓ RD↓
Rieckman et al.	2016	USA/ Harvard Aging Brain Study	M = 2.6 y	HC 108, 56%	66-87, M = 73.7	FA, MD, RD, AD	12 regions: SLF, superior frontal occipital, IFOF, ACR, SCR, PCR, IC, GCC, BCC, SCC, CING, parahippocampal CING	Intercept at 66 and M annual ∆% (without variability in ∆)	RD> MD> AD>FA↓, Right>Left,
Köhncke et al.	2016	Sweden/Swedish National Study of Aging	2.3 y	HC 37, 58%	88-88, M = 83.2	FA, MD	CST	M±SD, skewness, kurtosis for t1 and t2, t2- t1±SD, unstandardized effects for ∆ after controlling for age, edu, and sex.	FA↓ MD↑, RD↑, AD↑
Cao et al.	2016	China	1 y (12-week cog. training)	HC Control: 14, 38%	M = 70	FA, MD, RD, AD	WM skeleton: t2-t1	Maps of whole-WM comparisons, cluster size, and peak p value	FA↓ MD↑
				HC RCT: 34, 36%	M = 69				
Burzynska et al.	2017	USA/Fit and Active Seniors	6 m (RCT, walking, dance, nutrition)	Total: 174, 69%	60-80	FA, MD, RD, AD	CING, ALIC, Fmaj, Fmin, FX, gyrus rectus, HIPP, ILF, IFOF, PCC, PLIC, 6 subsections of CC, UN, PFC, whole WM	%∆ M±SD	HC: FA↓, RD↑, MD↑, AD↑
				HC Control: 40, 68%	M = 65				
De Lange et al.	2017	Norway/ Neurocognitive Plasticity	10 weeks (RCT, memory training)	HC: 49	M = 73.4	FA, MD, RD, AD	Voxelwise on WM skeleton	MD (plots only), only group x time interaction, no statistics for longitudinal ∆DTI reported	FA↓, RD↑, MD↑, AD↑
				HC: 28	M = 26.1				
Song et al.	2018	USA/Dallas Lifespan Brain Study (DLBS)	4 y	HC 52, 73%	55-89, M = 70.7	FA, RD, AD	parahippocampal CING, FX, whole WM	t1 and t2 RD in FX (on plots), annual change rate (R^2^, p), β and SE for annual ∆ rate	Not reported (mentioned FA↓, RD↑, MD↑, AD↑ in control but unclear if it was significant)
Benitez et al.	2018	USA	M = 15.2 m	HC 39, 72%	60-80, M = 67.7	FA, MD	Regions on WM skeleton: GCC, BCC, SCC, CP, CST, CING, FX, PLIC, SLF, SS, SFOF; UN	Annual %∆ M±SD	FA↓, MD↑
Staffaroni et al.	2018	USA	2.9 y	HC 69, 58%	61-87, M = 71.7	FA, MD	FX	Annual ∆ (β±95 CI)	FA↓, MD↑
Williams et al.	2019	USA/Baltimore Longitudinal Study of Aging	3.6 y	HC 406, 58%	50-95, M = 71.3	FA, MD	Regions: SLF, SFO, IFOF, SS, CING gyrus and hippocampus, GCC, BCC, SCC, ACR, SCR, PCR, ALIC, PLIC	Annual ∆ (β±95 CI and t-values from LME)	FA↓ MD↑
Kocevska, Cremers, et al.	2019	Netherlands/Rotterdam Study	5.2 y (2.8-8.1 y)	HC 2125, 56	45-87, M = 56	FA, MD	Global WM and tracts: Brainstem, CST, ATR, STR, PTR, SLF, ILF, IFOF, UN, CING gyrus, CING parahippocampus, FX, Fmin, Fmaj	Global WM FA and M±SD at t1 and t2	No ∆ in FA, MD
Nicolas et al.	2020	France/Agrica MSA IFR de Santé Publique	3.4 y	HC 130, 47APOE e4+, 27	65-85, M = 74.1	MD	Whole WM on WM skeleton	t2-t1 (M±SD)	↑MD
Beck et al.	2021	Norway/Tematisk Område Psykoser and StrokeMRI	1.2 y	HC 258, 33%	18-95, M = 55.6	FA, MD, RD, AD	Voxelwise for whole WM	Fixed effect of time (β±SD) predicted ∆ with age plotted as derivative values.	FA↓, RD↑, MD↑, AD↑
Coelho et al.	2021	Portugal/Switchbox consortium	4.3 y	HC 51, 51%	51-82,	FA, MD, RD, AD	Regions were organized in clusters of ROIs that varied by DTI metric.	Annual %∆ and slopes by clusters of ROIs not by individual ROI.	FA↓ MD↑, AD↑
					M = 63.5				

Only information relevant for DTI is included (other diffusion metrics or volumetric data are not reported). AD: axial diffusivity, ALIC: anterior limb of internal capsule, ARC: arcuate fasciculus, BCC: body corpus callosum, BP: blood pressure, BMI: body mass index, CC: corpus callosum, CING: cingulum, CP: cerebral peduncles, CST: corticospinal tract, Δ: change, EC: external capsule, FA: fractional anisotropy, Fmaj: forceps major, Fmin: forceps minor, FX: fornix, GCC: genu corpus callosum, HC: healthy controls, IFOF: inferior frontal-occipital fasciculus, IC: internal capsule, ILF: inferior longitudinal fasciculi, LME: linear mixed effect, M: month, MCI: mild cognitive impairment, MD: mean diffusivity, PCC: posterior cingulate cortex, RD: radial diffusivity, SCR: superior corona radiata, SLF: superior longitudinal fasciculus, SFOF: superior frontal-occipital fasciculus, t1: time point 1, t2: time point 2, UN: uncinate fasciculus, WMH: white matter hyperintensities, y: year.

### 2.6 Meta-analysis

#### 2.6.1 Effect size estimation

Our meta-analyses focused on FA and two regions of interest: whole WM (*n* = 12) and genu of the corpus callosum (*n* = 9), as these regions allowed us to include the largest number of studies. We included the splenium of the corpus callosum (*n* = 4) for exploratory analyses. We did not include MD, RD, AD, or other WM regions as insufficient number of studies overlapped in reporting these DTI metrics and WM regions (Table 1).

We used the R package “metafor” to estimate the mean and standard deviation of the distribution of the outcome effect size using a random-effects model (Viechtbauer, 2010). For our effect size, we calculated Cohen’s *d* or standardized mean difference (SMD) as the difference between two means (i.e., post-pre time measures), standardized by the pooled within-sample estimate of the population SD, calculated as SD (pooled within-sample) = $\sqrt{\frac{SD1^{2}+SD2^{2}}{2}}$ where SD1 is the standard deviation for the baseline measurement and SD2 is the standard deviation for the follow-up measurement. We calculated the standard error of the SMD with the formula $\text{SE}=\sqrt{\left(\frac{1}{N}\right)+\left(\frac{SMD^{2}}{2N}\right)x}\sqrt{2\left(1-Corr\right)}$ which accounts for the covariance between the two measurements and provides a more accurate estimate of the precision of the SMD, as recommended in the Cochrane Handbook (Section 23.2.7.2).

#### 2.6.2 Heterogeneity analysis

We estimated heterogeneity using the I² statistic, which represents the percentage of variance between studies attributable to differences in true effect sizes across studies rather than sampling variability. Although there is no universal threshold for interpreting the I², values of 25%, 50%, and 75% are commonly used to denote low, moderate, and high heterogeneity, respectively. However, I² estimates may be imprecise because they are influenced by the precision of the individual study effect sizes and the presence of outliers (Ioannidis et al., 2007). To address this potential issue, we calculated 95% confidence intervals for the I² estimate using the Q-profile method (Viechtbauer, 2007).

Heterogeneity variance was calculated using the restricted maximum likelihood (REML) method (Langan et al., 2019). To further explore the heterogeneity of the effect sizes and the robustness of our meta-analysis, we employed Graphical Display of Study Heterogeneity (GOSH) plots (Olkin et al., 2012) to display the effect sizes across studies. We then employed three supervised machine learning (k-means, DBSCAN, and the Gaussian Mixture Model) algorithms to detect clusters in the GOSH plot data and identify outlying and influential studies in our data. Lastly, to examine the potential for publication bias, we performed funnel plots and Egger’s regression tests for funnel plot asymmetry.

#### 2.6.3 Regions of interest for the meta-analyses

Whole WM FA was calculated as a mean of all regions-of-interest for the six studies (Barrick et al., 2010; Bender, Völkle, et al., 2016; Lövdén et al., 2014; Rieckmann et al., 2016; Storsve et al., 2016; Voss et al., 2013), whereas the other six-studies provided mean FA values for the whole WM using skeletonized data derived from Tract-Based Spatial Statistics (Beck et al., 2021; Burzynska et al., 2017; de Lange et al., 2017; Kocevska, Cremers, et al., 2019; Staffaroni et al., 2018; Teipel et al., 2010). Similarly, we included nine studies in the corpus callosum meta-analysis; we used data from the forceps minor for three studies (Lövdén et al., 2014; Storsve et al., 2016; Teipel et al., 2010). For the splenium, we included four studies in the meta-analysis (Bender, Völkle, et al., 2016; Burzynska et al., 2017; Rieckmann et al., 2016; Teipel et al., 2010).

#### 2.6.4 Analysis of modifiers of change using individual-level data

Lastly, we performed linear mixed-effects models using the lme4 package in R for a subset of studies (*n* = 6 studies, *n* = 375 subjects) that provided individual FA data (Beck et al., 2021; Bender, Völkle, et al., 2016; Burzynska et al., 2017; Rieckmann et al., 2016; Teipel et al., 2010; Voss et al., 2013). We added a random intercept for study and fixed effects for time point, age, sex, time until follow-up and sex-by-age interaction. To create partially standardized regression coefficients, we standardized all quantitative variables, but not factors. All analyses were conducted in R version 4.0.1, and statistical significance was accepted at *p* < 0.05 for two-tailed tests.

## 3 Results

### 3.1 Within-person changes in DTI parameters— a qualitative summary

To provide a qualitative summary of within-person changes in DTI parameters, we analyzed 30 studies included in our systematic review (Table 1). FA was the most frequently reported metric (29 studies) and in 77% studies (n = 23) there was a decline in FA. Notably, earlier studies (i.e., published 2009–2014) tended to report no significant changes in FA. MD was the second most reported metric (19 studies), and 53% (n = 16) reported an increase in MD. The less commonly reported metrics were RD (18 studies) and AD (16 studies) were less commonly reported, with 43% (n = 13) and 33% (n = 10) reported increases in RD and AD, respectively. Table 2 provides a visual summary of the changes in each DTI parameter.

**Table 2.**
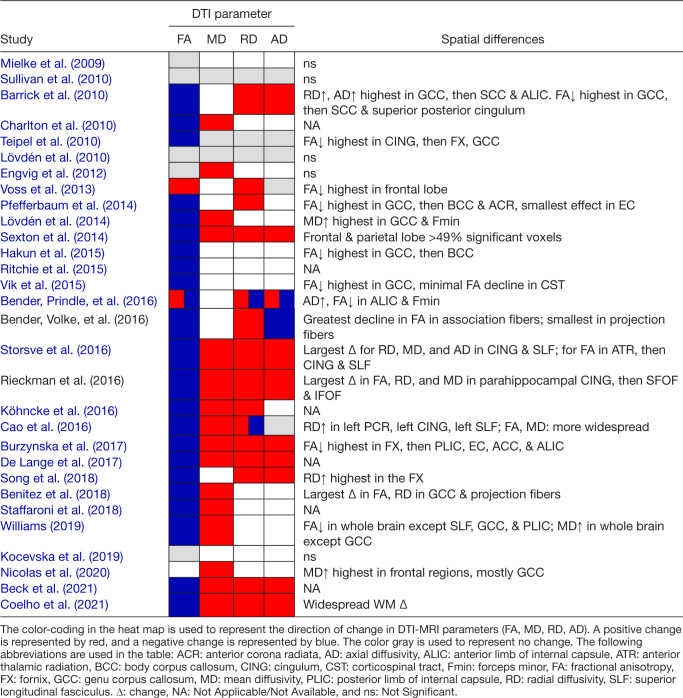
Within-person change in DTI metrics, moderators of change, and regional differences.

### 3.2 Within-person changes in FA of the whole WM—a meta-analysis

**Fig. 3.**
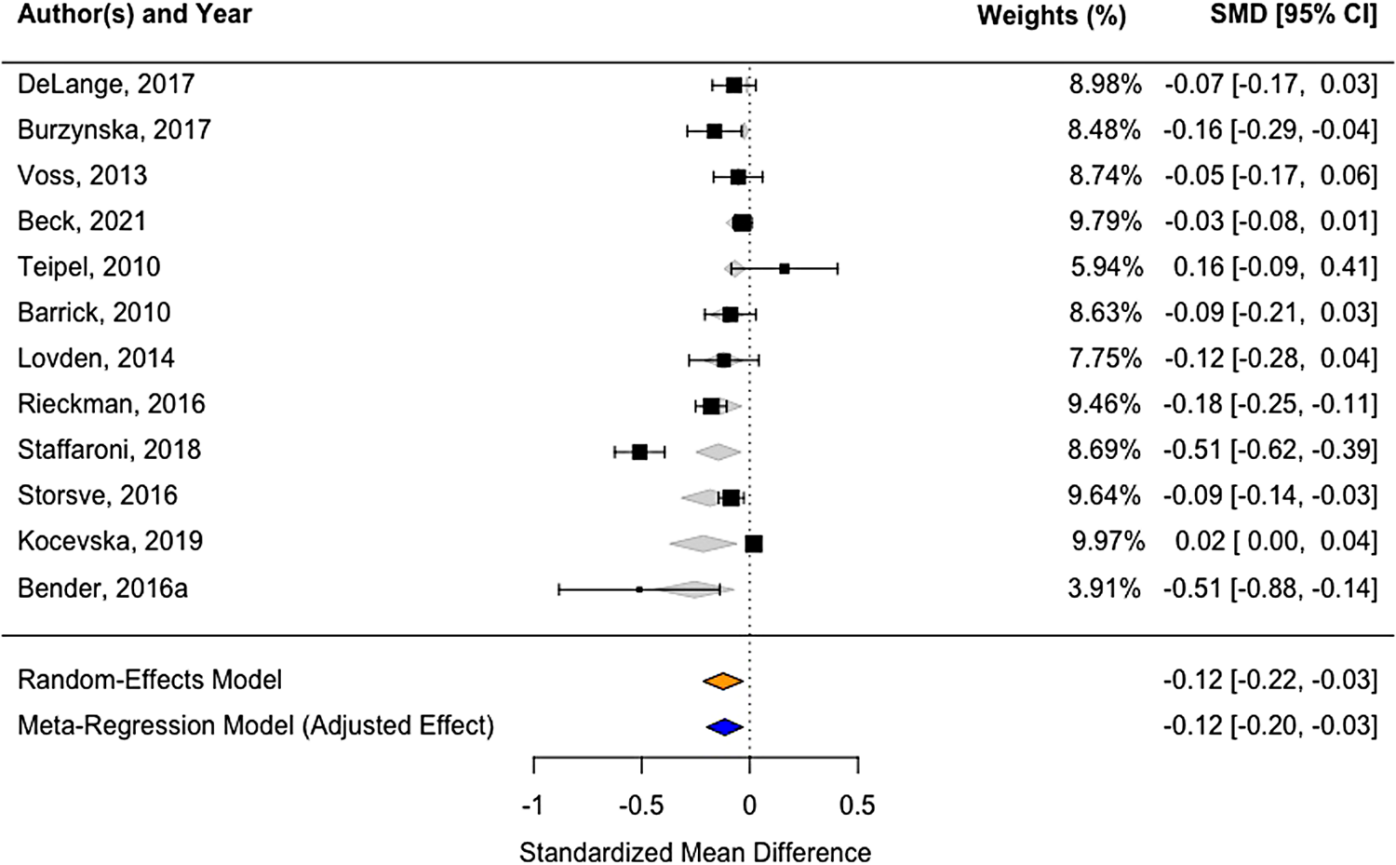
Forest-plot showing standardized effects sizes of FA decline in whole WM using summary statistics across 12 studies. Box size represents study weights. At the bottom, we display final summary estimates with 95% CI for unadjusted vs. adjusted models (accounting for study follow-up time as a moderator). The weights for each study are calculated as the inverse of the variance of the effect size estimate for the study, meaning that the larger the standard error of an effect size estimate, the smaller the weight.

To account for the different ways effect sizes were reported across studies, we selected a subset of studies that provided sufficient data to calculate the *d*-statistic defined as the change between two timepoints divided by pooled standard deviation. For the whole WM, we obtained data from 12 studies (Fig. 3). The pooled effect showed a significant decline in the whole WM FA (*d* = -0.1235, 95% CI: -0.21 to -0.03, *p* = 0.0086), both when adjusted and not adjusted for the follow-up time as a moderator. Heterogeneity across the studies was substantial (I² = 93.5% after adjusting for study follow-up time as a covariate).

**Fig. 4.**
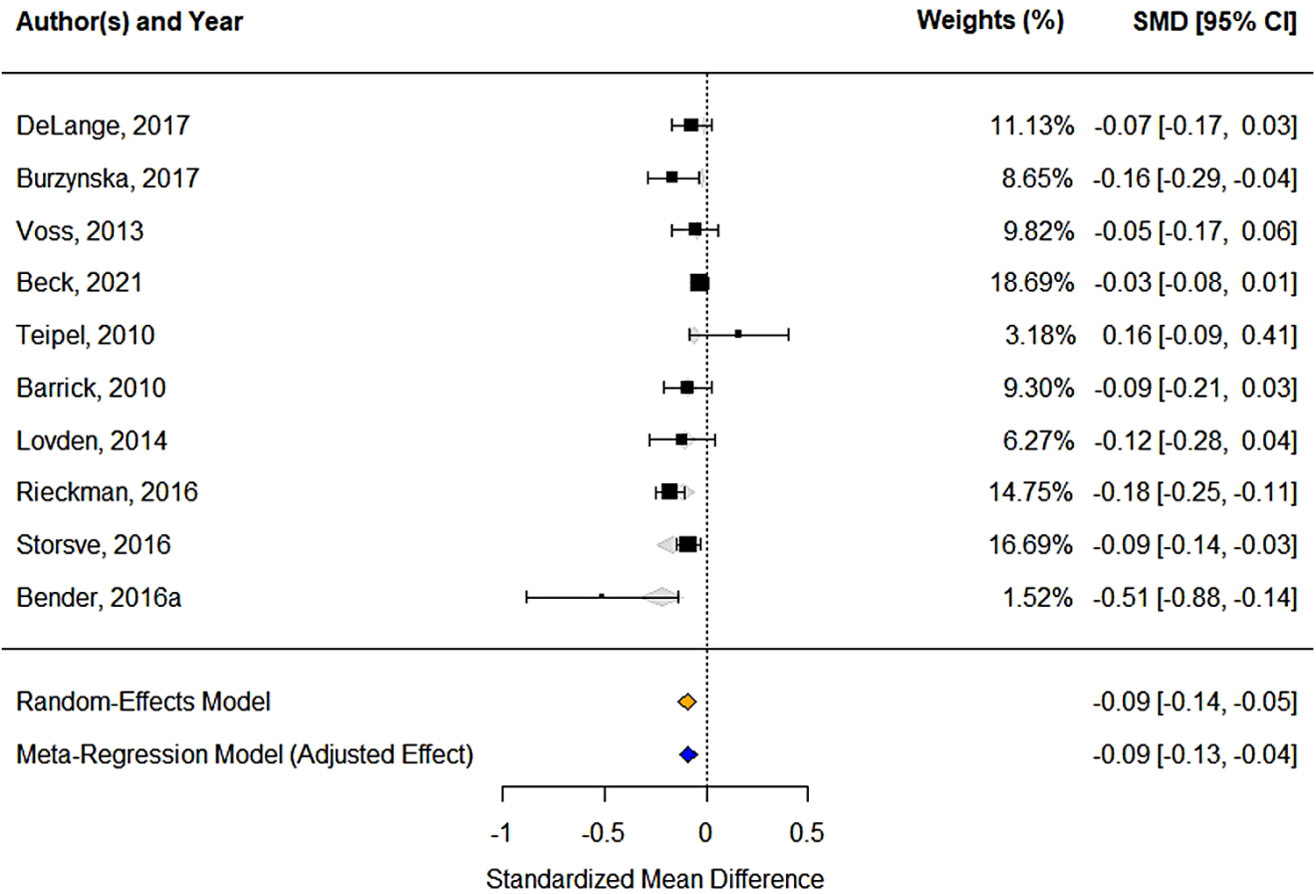
Forest-plot showing standardized effects of FA change across in the whole WM using summary statistics across 10 studies (after omitting two outlier studies). Box size represents study weights. At the bottom, we display final summary estimates with 95% CI for the random-effect model. Removed as outliers: Staffaroni et al., 2018 and Kocevska, Cremers, et al., 2019. The weights for each study are calculated as the inverse of the variance of the effect size estimate for the study, meaning that the larger the standard error of an effect size estimate, the smaller the weight.

To address the high heterogeneity, we performed diagnostic testing for influential cases (outliers) with GOSH plots, followed by sensitivity analyses, which identified two outlier studies (Kocevska, Cremers, et al., 2019; Staffaroni et al., 2018). We repeated the random effects model without the two outliers, which confirmed the significant negative change in FA, but with reduced heterogeneity (residual I² = 48%); Figure 4 (see Table 3 for model comparisons).

**Table 3.** Meta-analysis of within-person declines in FA in the whole WM: comparison of the full model and with excluded influential studies.

Analysis	*N*	*Age*	*d*	95% CI	*p*	*I*²	95% CI of the *I*²
Main analysis (Fig. 3)	2906	66.4	-0.12	-0.21; -0.03	0.008	95%	88.7; 98.6
Influencing cases removed (Fig. 4)*	724	66.6	-0.09	-0.13; -0.04	<0.001	49%	12.54; 96.89

* Removed as outliers: Staffaroni et al. (2018) and Kocevska et al. (2019).

The reduction in heterogeneity indicates that approximately 48% of the total variance in FA can be attributed to heterogeneity among the studies, with the remaining 2% attributed to sampling variance. In sum, the model comparison indicated a robust and significant, yet small effect size of within-person declines in FA in the whole WM despite the heterogeneity observed among the studies.

### 3.3 Within-person changes in FA of the genu and splenium corpus callosum—a meta-analysis

**Fig. 5.**
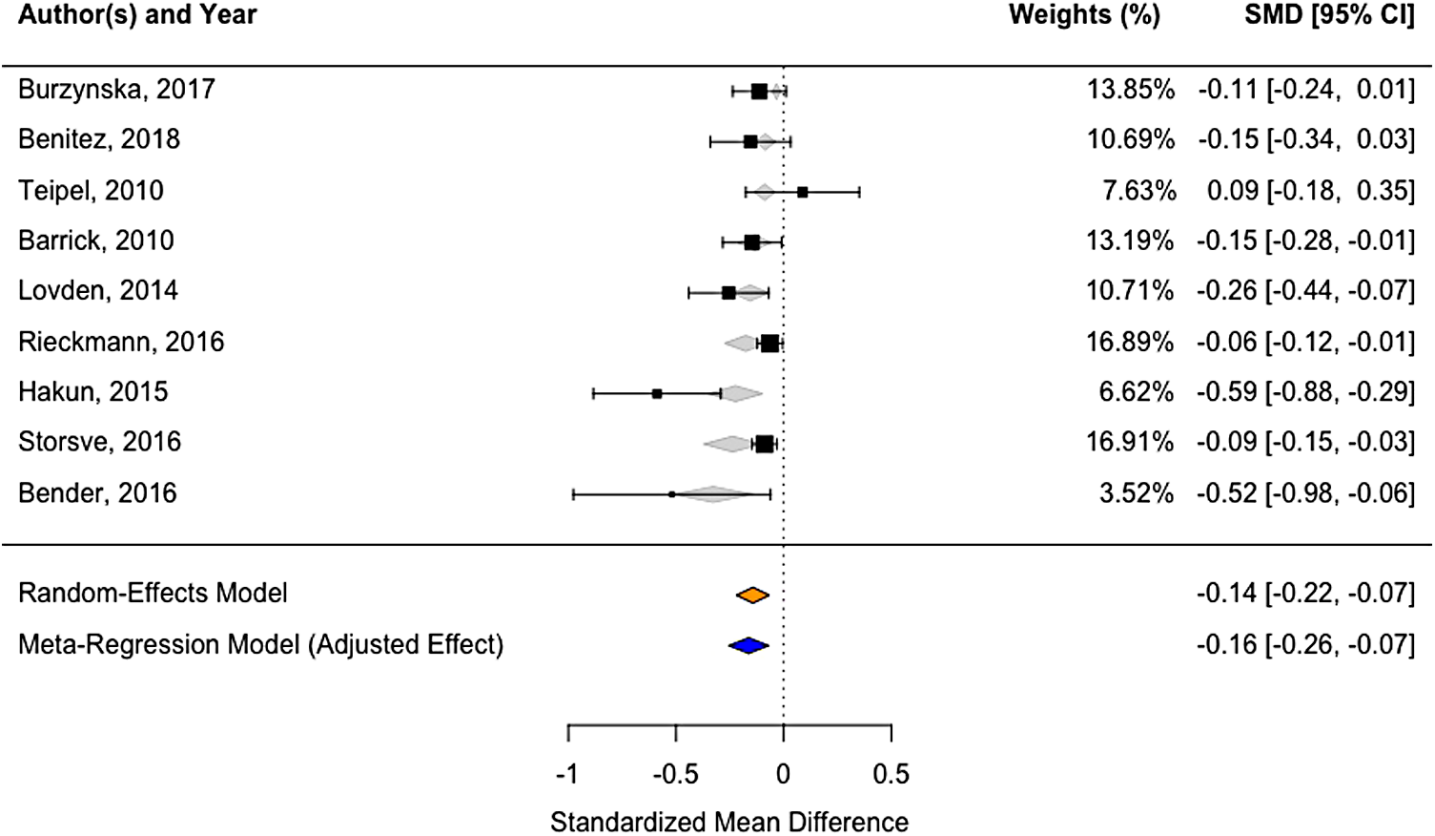
Forest-plot showing standardized effects of FA change in the genu of the corpus callosum across nine studies. Box size represents study weights. At the bottom, we display final summary estimates with 95% CI for unadjusted vs. adjusted models accounting for study follow-up time as a moderator. The weights for each study are calculated as the inverse of the variance of the effect size estimate for the study, meaning that the larger the standard error of an effect size estimate, the smaller the weight.

For the genu corpus callosum, we obtained data from nine studies. The pooled effect among 550 participants (69.2 ± 6.8 years old) showed a significant negative change in FA (*d* = -0.1432, 95% CI: -0.22 to -0.06, *p* = 0.0003, Fig. 5). We noted a moderate level of heterogeneity (residual I² = 65%, 95% CI: 30.65% to 97.58%), suggesting that the variability in the results could be influenced by specific differences across the individual studies.

**Fig. 6.**
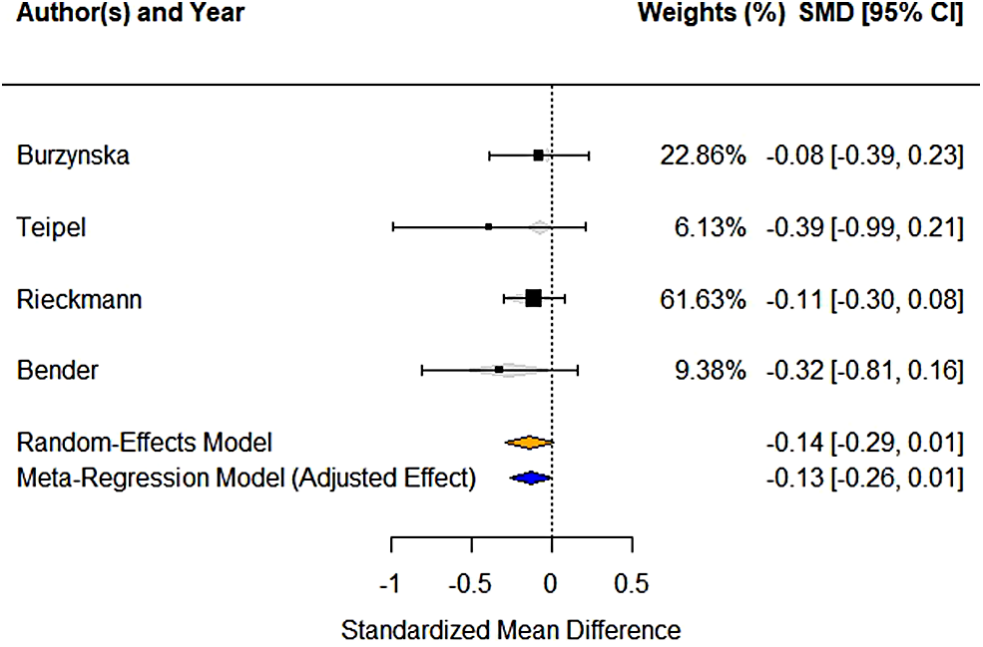
Forest-plot showing standardized effects of FA change in the splenium of the corpus callosum across four studies.

For the splenium corpus callosum, we obtained data from four studies. The pooled effect among 176 participants (67.7 ± 4.0 years old) showed a non-significant negative change in FA (*d* = -0.1399, 95% CI: -0.2881 to 0.0084, *p* = 0.0644, Fig. 6), with a level of heterogeneity (residual I² = 0). However, it is important to note the wide confidence interval for this I² estimate (0% to 90.10%), indicating a high degree of uncertainty about the true level of heterogeneity.

### 3.4 The effect of follow-up time on change in FA

**Fig. 7.**
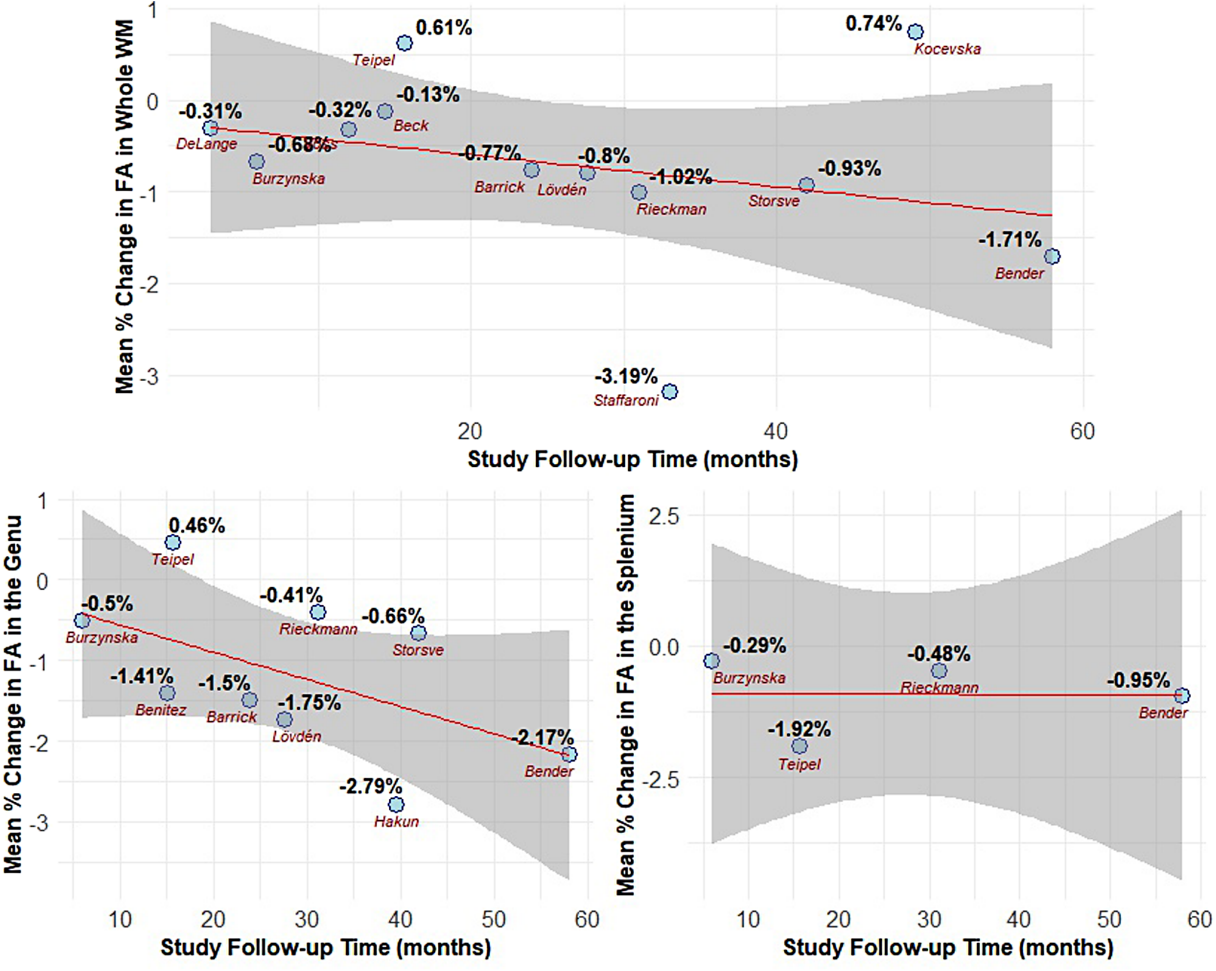
Mean study follow-up time was associated with greater decline in FA in the whole WM and genu (more negative mean % change). The regression lines represent the results of a linear model fitted to the data. The shaded area around the line represents the standard error. Points display the percent change for each study.

To understand the effect of follow-up time (i.e., the time elapsed between the two measurements) on FA change, we correlated the mean % change in the whole WM, genu, and splenium of the corpus callosum with the mean study follow-up time among the studies included in the meta-analyses. We found a trend towards increased decline in FA with longer follow-up times in the whole WM and genu (whole WM *r* = -0.28, 95% CI: -0.74 to 0.34, *p* = 0.361; genu of the corpus callosum *r* = -0.53, 95% CI: -0.88 to 0.19, *p* = 0.134; Fig. 7). We found no correlation between mean % change and follow-up time in the splenium, with a wide confidence interval indicating high uncertainty (*r* = -0.02, 95% CI: -0.96 to 0.96, *p* = 0.977).

### 3.5 The effect of age and sex on change in FA

To examine the effects of age (at the time of the first or baseline measurement) and sex on within-person changes in DTI parameters, we (1) conducted a qualitative analysis of the 14 studies that included age and sex as covariates in their analyses and (2) performed a quantitative analysis of the studies that provided individual FA data at both time points.

#### 3.5.1 Effects of age: qualitative analysis

Out of 14 studies reporting relevant data, 10 studies found that older age at baseline was associated with greater magnitude of decline in FA (Beck et al., 2021; Bender, Prindle, et al., 2016; Bender, Völkle, et al., 2016; Burzynska et al., 2017; Pfefferbaum et al., 2014; Sexton et al., 2014; Song et al., 2018; Storsve et al., 2016; Voss et al., 2013; Williams et al., 2019), while one study reported no effect of age on FA change (Barrick et al., 2010). Several studies associated older age with a greater increase in MD (Beck et al., 2021; Charlton et al., 2010; Engvig et al., 2012; Lövdén et al., 2014; Nicolas et al., 2020; Storsve et al., 2016; Williams et al., 2019), a greater increase in RD (Beck et al., 2021; Bender, Völkle, et al., 2016; Sexton et al., 2014; Song et al., 2018; Storsve et al., 2016), and a greater increase in AD (Beck et al., 2021; Bender, Prindle, et al., 2016; Bender, Völkle, et al., 2016; Sexton et al., 2014; Storsve et al., 2016).

Notably, two studies across the lifespan specifically reported an accelerated decline in FA after the fifth decade of life (Sexton et al., 2014; Storsve et al., 2016). Furthermore, Beck et al. (2021) showed that FA plateaued around the third decade, with a steady decline following the age of ~40 years and an accelerated decrease in older age. For MD, AD, and RD, these metrics decreased until the 40–50-year age mark and subsequently increased following a steady period. This pattern is consistent with previous cross-sectional data (Bartzokis et al., 2004; Lebel et al., 2012) and shows an inverted U-shape for FA and a U-shape for other DTI metrics, with an inflection point at approximately 40-–50 years of age.

#### 3.5.2 Effects of sex: qualitative analysis

Out of seven studies that investigated sex differences in within-person changes in DTI parameters (Beck et al., 2021; Burzynska et al., 2017; Nicolas et al., 2020; Sexton et al., 2014; Teipel et al., 2010), only two reported significant sex differences. Williams et al. (2019) found that women (aged 50–95 years) showed a greater decline in FA in the cingulum and a greater MD increase in the genu of the corpus callosum. In contrast, in a study of very old adults (aged 81–103 years), Lövdén et al. (2014) found that women had a smaller decline in FA in the forceps minor than men.

#### 3.5.3 Effects of age and sex: quantitative analysis in the whole WM

**Fig. 8.**
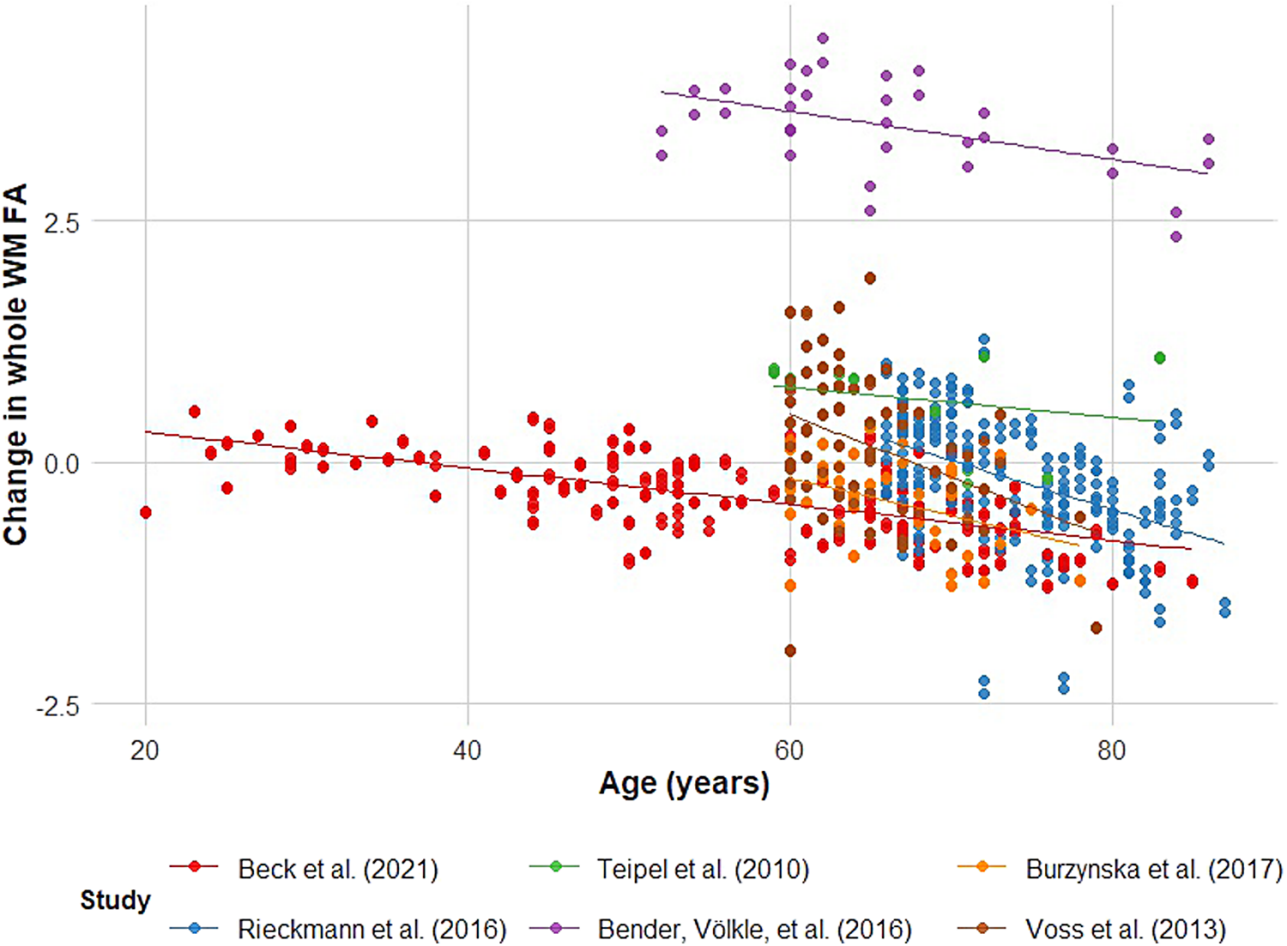
Older age correlated with more negative change in the FA of the whole WM. Each point represents an individual’s predicted FA change based on the linear mixed-effects analyses. The solid lines represent the linear regression line for each study.

A linear mixed-effects model using individual-level FA supplied to us by authors (Beck et al., 2021; Bender, Völkle, et al., 2016; Burzynska et al., 2017; Rieckmann et al., 2016; Teipel et al., 2010; Voss et al., 2013) showed that older age, female sex, and longer follow-up time were associated with greater declines in FA. We also observed an interaction between age and sex, with the negative effect of age on FA change being about 48% larger in females than in males (Table 4 and Fig. 8).

**Table 4.** Linear mixed-effects analysis of within-person change in the whole WM.

	Full model		
Model parameter	***β***	**SE**	***p***
Intercept	0.589	0.540	0.203
Age (baseline)	-0.237	0.042	0.001
Time until follow-up	-0.122	0.125	0.001
Sex	-0.189	0.056	0.001
Sex-by-age interaction	-0.114	0.055	0.002

The total number of observations were 750, consisting of 375 participants each measured at two time points: baseline and follow-up. Number of groups (random effect by studies): 6. Sex is coded as 0 for males and 1 for females. *β* are standardized. The model estimates the effects of various predictor variables on the change in FA of the whole WM over time, including age at baseline, time until follow-up, sex, and a sex-by-age interaction.

In subsequent exploratory analysis of individual-level FA data, greater baseline age and time until follow-up correlated with greater FA decline in both genu and splenium of the corpus callosum (Table 5).

**Table 5.** Linear mixed-effects analysis of within-person change in the corpus callosum.

Model parameter	Genu			Splenium		
	***β***	**SE**	***p***	***β***	**SE**	***p***
Intercept	0.777	0.782	0.429	0.504	0.369	0.267
Age (baseline)	-0.156	0.036	0.001	-0.205	0.048	0.001
Time until follow-up	-0.222	0.154	0.153	-0.344	0.153	0.033
Sex	0.057	0.048	0.240	0.133	0.065	0.042
Sex-by-age interaction	0.048	0.048	0.318	0.057	0.016	0.490

For the genu: We recorded 165 participants, consisting of 330 observations at baseline and follow-up across 3 study groups. For the splenium: We documented 176 participants, consisting of 352 observations at two time points across 4 study groups.

### 3.6 Spatial patterns of within-person changes: qualitative summary

Due to the wide variability in defining regions of interest among the 30 studies in Table 1, we could not directly compare the effect sizes of FA change across different regions in a meta-analysis. Thus, we offer a qualitative summary of our findings (see Table 2).

In brief, only three studies claimed to observe the development-to-degeneration pattern of WM decline (Bender, Prindle, et al., 2016; Bender, Völkle, et al., 2016; Storsve et al., 2016). However, a systematic review of the remaining studies indicated that, in general, there were greater changes in FA, MD, and RD in late-myelinating regions, such as the genu of the corpus callosum, anterior limb of the internal capsule, and fornix, than in early myelinating regions, such as the superior corona radiata, posterior limb of the internal capsule, and corticospinal tract (Barrick et al., 2010; Bender, Völkle, et al., 2016; Teipel et al., 2010; Vik et al., 2015) (Table 2). Specifically, several studies reported the largest within-person changes in the genu of the corpus callosum (Barrick et al., 2010; Benitez et al., 2018; Hakun et al., 2015; Lövdén et al., 2014; Nicolas et al., 2020; Pfefferbaum et al., 2014; Teipel et al., 2010; Vik et al., 2015). The largest within-person change in the anterior corpus callosum was more evident in studies with mean ages ranging from 59 to 68 years (Barrick et al., 2010; Benitez et al., 2018; Teipel et al., 2010; Vik et al., 2015), whereas studies examining adults aged 70 years or older tended to observe more pronounced changes in DTI parameters in early myelinating regions, such as the corticospinal tract, the superior, and posterior corona radiata (Köhncke et al., 2016; Lövdén et al., 2014; Rieckmann et al., 2016). In contrast, three studies showed no evidence of spatial gradients in WM change (Cao et al., 2016; Coelho et al., 2021; Williams et al., 2019).

### 3.7 Other modifiers of within-person changes in DTI parameters

This section highlights lifestyle, health, and genetic modifiers and correlates of changes in DTI parameters in adult WM investigated among the 30 studies (Table 1).

#### 3.7.1 Health-related modifiers

##### 3.7.1.1 General health indicators

Telomere attrition was associated with greater FA decrease and MD increase in the fornix, even after controlling for physical activity and vascular risk (Staffaroni et al., 2018). In contrast, sleep duration and quality were not related to DTI changes (Kocevska, Cremers, et al., 2019).

##### 3.7.1.2 Cardiovascular risk factors

Higher baseline cumulative burden of vascular risk factors (i.e., hypertension, obesity, elevated cholesterol, diabetes, and smoking status) was associated with greater decline in FA in the parahippocampal cingulum, fornix/stria terminalis, and splenium of the corpus callosum and greater increases in MD in the splenium of the corpus callosum in otherwise cognitively healthy older adults (Williams et al., 2019). Another study reported trend-level associations between diagnosed hypertension and greater within-person increase in AD and RD (Bender, Völkle, et al., 2016).

#### 3.7.2 Lifestyle modifiers

##### 3.7.2.1 Physical activity and social activities

A 6-month dance intervention led to a slower decline in FA and smaller RD increase in the fornix compared to control and aerobic walking groups, and spending less time sedentary and engaging more in moderate-to-vigorous physical activity at study baseline correlated with lesser 6-month decline in prefrontal FA (Burzynska et al., 2017). Notably, adding a nutritional supplement (beta alanine) to walking did not seem to affect within-person changes in WM compared to walking alone. Another 1-year RCT with the same aerobic walking intervention group reported no group-level level effects of exercise, but found that greater aerobic fitness gain correlated with more positive FA changes in the frontal and temporal lobes (Voss et al., 2013). Engagement in social leisure activities over a 3-year period was associated with increased FA in the corticospinal tract and improved processing speed in individuals older than 80 years (Köhncke et al., 2016). In sum, physical activity and aerobic fitness is a promising protective lifestyle factor for allowing down or reversing age-related FA declines.

Interestingly, studies considering genetic risk of Alzheimer’s disease have reported mixed results: engaging in physical activity was associated with greater increases in MD and AD among healthy adults with APOE ε4 genotype (Raffin et al., 2021) and increased MD in patients with subjective cognitive impairment (Maltais et al., 2020). These findings suggest that the effects of physical activity on WM changes may vary in various clinical groups and warrants further investigation.

##### 3.7.2.2 Cognitive training

Similarly, cognitive training has inconsistent effects on within-person change in FA. Engvig et al. (2012) reported a reduced decline in FA in the anterior WM after 12 weeks of memory training compared to controls. Lövdén et al. (2010) documented an increase in FA in the genu for older, but not younger participants, following a 100-hour cognitive training. Similarly, De Lange et al. (2017) found that older adults in the cognitive training group experienced less age-related decline in FA and a smaller increase in MD, RD, and AD compared to the control group in areas, including the corpus callosum and the cortico-spinal tract; these effects were not observed in younger participants. Other 12-week cognitive training interventions failed to find any changes in within-person change (Cao et al., 2016; Lampit et al., 2015).

##### 3.7.2.3 Alcohol consumption

Heavy-drinking relapsers showed a steeper decline in FA compared to abstainers, in areas such as the anterior commissural tracts (genu and body), projection fibers (corona radiata, external capsule, internal capsule anterior limb), and association fibers (superior longitudinal fasciculus)(Pfefferbaum et al., 2014).

#### 3.7.3 Genetic risk and neurological modifiers

##### 3.7.3.1 Genetic risk and biomarkers of Alzheimer’s disease

APOE ε4 carriers had a significantly greater decline in FA in the genu and body of the corpus callosum and splenium of the corpus callosum compared to non-carriers, but did not differ in rates of change in MD (Williams et al., 2019). Healthy older adults with a higher amyloid burden showed accelerated FA decline in the parahippocampal cingulum, body corpus callosum, and forceps minor than those with low amyloid burden, even after controlling for hippocampal atrophy (Rieckmann et al., 2016). Other biomarkers such as YKL-40 and amyloid-beta have also been found to be predictive of greater within-person changes in MD (Racine et al., 2019) and RD (Song et al., 2018).

##### 3.7.3.2 Cognitive status

Fletcher et al. (2013) have reported that greater within-person changes in AD in the fornix were associated with an increased risk of conversion to mild cognitive impairment in healthy older adults, whereas others found no magnitude differences but greater inter-person variability in FA change between participants with mild cognitive impairment compared to the healthy controls (Teipel et al., 2010).

## 4 Discussion

Our meta-analysis and longitudinal models demonstrated that:

- WM microstructure undergoes significant changes throughout adulthood.
- Within-person changes can be captured non-invasively using DTI.
- The pooled effect size of FA declines was *d* = -0.12 in whole WM, *d* = -0.16 in genu, and *d* = -0.13 in the splenium of the corpus callosum.
- The magnitude of within-person changes increases with advancing age in the whole WM, genu, and splenium of the corpus callosum.
- Female sex is associated with increased decline in the whole WM.
- Longer follow-up times were associated with increased decline in FA in the whole WM and genu.
- Regarding the spatiotemporal pattern of changes in WM, evidence for the anterior-to-posterior gradient in the corpus callosum remains inconclusive.

The outcomes of the systematic review suggest that:

- Changes observed in the adult WM include predominantly declines in FA, increases in MD and RD, and to a lesser extent, increases in AD.
- Changes in WM can be detected within a relatively short period of 6 months. However, results are more consistent when the follow-up time is longer.
- The within-person changes in DTI parameters tend to follow the development-to-degeneration spatiotemporal pattern, with greater magnitude of change in late-myelinating regions.
- There is promising evidence for protective effects of physical activity and social activities against age-related FA change, with inconclusive effects of cognitive training.
- Other modifiers of individual change in DTI parameters include sex, hypertension, and presence of biomarkers for Alzheimer’s disease.
- Heavy alcohol consumption, vascular risk factors, and carrying the APOE ε4 allele are potential risk factors for accelerated FA decline.

Below, we discuss main findings, limitations, and recommendations for future longitudinal studies on WM aging.

### 4.1 Main findings and challenges of the current analyses

#### 4.1.1 Magnitude of change

We found that the magnitude of within-person changes increased with advancing age. However, we could not calculate an effect size estimate per year because of varying follow-up times across studies (ranging from 2 to 58 months), as only two studies had a 12-month follow-up duration (Cao et al., 2016; Voss et al., 2013). We did not standardize our effect size estimates per year, as this would require a strong assumption of a common linear change in WM across all studies. Given the large heterogeneity in the effect sizes across studies, we do not think this assumption is reasonable. Therefore, it is important to acknowledge the limitations of our current understanding of the rate of decline in FA in WM in aging individuals, given the variability in follow-up durations and potential nonlinear trajectories of change. Future studies with more uniform follow-up durations would be needed to estimate the effect size of this decline more accurately.

#### 4.1.2 Is there regional variability in WM changes?

Our systematic review supports the notion of selective vulnerability to aging and neurodegeneration in late-myelinating WM regions, such as the fornix and genu of the corpus callosum (Lacalle-Aurioles & Iturria-Medina, 2023; Raghavan et al., 2020). Our meta-analysis showed a negative change in FA in the genu and splenium of the corpus callosum with comparable effect sizes. The fact that the changes were significant in the genu but not in the splenium may be due to differences in statistical power between the two analyses (n = 550 vs. n = 176), leaving the evidence for selective vulnerability in the anterior corpus callosum inconclusive. Considering the range of sample sizes in our included studies—spanning from 11 to 108 for the splenium and 11 to 201 for the genu—it is prudent for future research to calibrate their sample sizes informed by our findings. A larger sample size may reveal greater effect sizes in anterior commissural zones like the genu, aligning with current theories of aging.

While our review did not aim to study WM lateralization, select studies, like Cao et al., 2016, observed within-person change primarily in the left cingulum and superior longitudinal fasciculus. Additionally, Ritchie et al. found that including the correlation between bilateral tracts improved a single-factor cognition model. However, there is a possibility that variance in the FA changes of specific tracts may provide additional information about cognitive decline (Ritchie et al., 2015). Future studies should directly examine WM lateralization in a longitudinal healthy aging sample.

#### 4.1.3 Effect of follow-up time and time periods to detect within-person change in WM

In the whole WM and corpus callosum, the meta-analyses and linear-mixed effects models showed a significant effect of follow-up time on changes in FA. Notably, adjusting for follow-up time as a moderator in the meta-analysis had a minimal effect on the pooled effect size estimates. It is possible that including studies with varying follow-up durations (6–58 months) introduced heterogeneity that affected the overall effect size estimate. Nevertheless, our findings suggest that a longer duration between MRI measurements is associated with a greater decline in FA.

However, in the splenium region, despite a significant main effect of follow-up time in the linear mixed-effects model, we did not find a correlation between average follow-up time and FA percent change by study. This lack of correlation may be attributed to lower statistical power, as the meta-analyses and correlational analyses used aggregated effect sizes, whereas the linear mixed-effects models employed data at the individual level.

In our qualitative review, we found that earlier studies with shorter follow-up times and small sample sizes did not find significant within-person changes in WM, while more recent research has reported small yet significant effects at shorter follow-up times. Studies with follow-up times ranging from 6- to 58 months consistently reported a decline in FA and increases in MD, RD, and AD over time, with a few exceptions (Kocevska, Cremers, et al., 2019) or mixed findings (Bender, Prindle, et al., 2016; Cao et al., 2016). This suggests that follow-up times shorter than 6 months might be insufficient to robustly detect within-person changes in WM microstructure in healthy adults, especially when sample sizes are limited. It is possible that the lack of significant within-person changes observed in the first DTI longitudinal studies can be attributed to the lack of standardization in DTI preprocessing pipelines and lower quality of diffusion sequences (Lövdén et al., 2010; Mielke et al., 2009; Sullivan et al., 2010). However, it is plausible that advancements in data processing techniques and the enhancement of artifact and noise removal methods (e.g., susceptibility or motion) have increased the sensitivity of DTI data to within-person changes.

#### 4.1.4 Modifiers of within-person change

Our systematic review suggests that aerobic exercise and cognitive training may have subtle effects on WM changes as measured by DTI (Burzynska et al., 2017; Engvig et al., 2012; Voss et al., 2013).

We also found that genetic factors such as the APOE ε4 allele and biomarkers for Alzheimer’s disease pathology (amyloid-beta) and chronic inflammation (YKL-40) appear to be promising modifiers of within-person WM changes (Racine et al., 2019; Song et al., 2018), especially in the fornix, a WM region susceptible to aging and neurodegeneration (Lacalle-Aurioles & Iturria-Medina, 2023). Additionally, Fletcher et al. (2013) noted that greater within-person changes in WM in the fornix predicted conversion to mild cognitive impairment. Similarly, greater within-person declines were predictive of decreased executive function, but not memory, in those with late mild cognitive impairment and dementia (Scott et al., 2017).

Despite the evidence that exercise, physical activity, and increased social engagement have an impact on WM health, many important questions remain to be answered. From a practical perspective, we still need to learn how to design exercise interventions that mitigate WM decline. Future research might be able to answer questions such as: When is it best to begin lifestyle interventions? Can exercise that combines cognitive stimulation with social interactions (e.g., dancing) have more positive effects on WM? How do social and environmental interactions relate to modifiers of WM health? What types of exercise work best for people with comorbidities such as diabetes and hypertension?

Regardless, we observed subtle and inconclusive findings of modifiers of within-person change in several studies, possibly due to varying follow-up times and study protocols.

### 4.2 Heterogeneity among studies

Heterogeneity among the MRI studies included in this review and meta-analysis impacts the observed effect sizes in within-person changes in white matter DTI over time. We identified several sources of heterogeneity. First, there is inconsistent reporting, such as the absence of mean and variances at baseline or follow-up, presenting only means at baseline, or providing latent change scores adjusted by other covariates without the corresponding raw mean scores or unadjusted estimates. The variations in the statistical measures reported (e.g., M±SE, M±SD, median±SD, β) further contributed to the challenges in comparing and synthesizing the results across the 30 studies (see Table 1 for more details). Moreover, we observed that most studies incorporated distinct regions of interest and often lacked consistent data on modifiers of healthy aging, such as hypertension and lifestyle factors. This restricted our capacity to execute a meta-analysis with higher statistical power and impeded our efforts to quantitatively assess the influence of these modifiers on within-person changes in WM.

Second, there were significant methodological differences in acquisition and processing of DTI data. For example, although the great majority of the studies used the TBSS procedure designed to minimize the effects of anatomical differences in samples with wide age ranges, several studies implemented various customizations of TBSS processing (Bender, Prindle, et al., 2016; Bender, Völkle, et al., 2016; Coelho et al., 2021; Engvig et al., 2012). Other studies employed alternative approaches such as manual drawing of ROIs and other semi-automated segmentation methods to extract subregions of the corpus callosum (Charlton et al., 2010; Lövdén et al., 2010; Mielke et al., 2009; Song et al., 2018; Staffaroni et al., 2018; Williams et al., 2019), or tractography-related methods (Kocevska, Cremers, et al., 2019; Storsve et al., 2016; Sullivan et al., 2010; Vik et al., 2015). Different ways to extract regional DTI data may affect not only the mean values of the DTI metrics, but also the sensitivity to change. For example, skeletonized data are extracted from the center of tracts, which omit areas of lower FA due to partial volume or white matter lesions, both of which may be confounded by aging. Consequently, the skeletonization method tends to overestimate FA values and underestimate the values of MD, RD, and AD compared to methods such as manual ROI delineation or atlas-based segmentation, which consider the whole tract.

Notably, DTI, mainly when processed using the standard TBSS pipeline, has shown robust stability over short periods of time. TBSS studies have indicated a low test-retest variability in DTI metrics with less than 0.4% mean differences and below 1.2% variance in measurements across and within scanner locations and a between-site intraclass correlation coefficient (ICC) exceeding 0.80 (Melzer et al., 2020). Similarly, a multisite study of healthy older adults found that FA had the strongest test-retest reliability with reproducibility errors consistently within 2-4% range (Jovicich et al., 2014). Another study of healthy older adults comparing conventional DTI measures to free water elimination diffusion MRI found that irrespective of the diffusion analysis method used, FA was the most reliable metric (ICC: 0.87 ± 0.05), while MD was the least reliable (ICC: 0.81 ± 0.09) (Albi et al., 2017).

However, we recognize that different processing pipelines can introduce variability, and the reliability of TBSS can vary by region and registration quality across images. As a step forward, we recommend future longitudinal studies, especially those employing varied diffusion models or preprocessing methods, to incorporate an evaluation of test-retest reliability for a subset of participants at baseline. Thus, while our reviewed studies suggest that longitudinal changes in DTI primarily arise from aging, not measurement error, the implications of methodological differences on within-person WM remain unresolved and would be an interesting topic for future investigation.

Lastly, given that probabilistic tractography can have varying degrees of reproducibility and reliability (Maier-Hein et al., 2017) and TBSS-ROI in standard space has shown excellent precision and reproducibility (Cai et al., 2021), we suggest that TBSS-ROI should be the method of choice for comparing and pooling results for future meta-analyses. In summary, our results show consistent negative changes in FA despite heterogeneity in DTI protocols and analyses; however, we could not determine how each potential source of heterogeneity influenced the pooled effect size in our analyses.

### 4.3 Recommendations for design and reporting in future longitudinal studies on WM

Based on the challenges we encountered in conducting meta-analyses, we suggest that future studies should strive to:

- Standardize the reporting of results. This should include, at a minimum, (a) parameter estimates for within-person changes in DTI, (b) standard deviations, (c) standard errors, and (d) pre- and post-measurement mean values. This information should always be included, for example, in Supplementary Materials.
- Report effect size estimates and 95% confidence intervals rather than *p*-values only.
- Consistently provide information on (a) mean follow-up times, (b) age at baseline and follow-up, (c) sample size at baseline and follow-up, and (e) correlations between pre- and post-measurements.
- Report null findings when appropriate.
- Use the standard TBSS-ROI procedure, at least as a point of reference. In doing so, use a study-specific “mean FA” and “skeleton” (rather than a standard template).
- Use a minimum follow-up time of 6 months to detect significant within-person changes in WM microstructure in healthy adults, especially when sample sizes are small.
- Include a properly designed control group when conducting an RCT. A crossover design, although beneficial for controlling for between-person variability, would not allow observation of the effects of time on natural within-person changes in WM.
- In both case-control studies and RCTs, report not only the effect of the intervention on within-person changes in WM but also the changes observed within the control groups.
- Acquire more data-rich datasets, such as using more diffusion-weighted directions and b = 0 images since increased quality of the diffusion sequence can lead to higher reproducibility of FA and MD in older adults.
- When using plots to present the changes in WM over time at different time points instead of tables, include mean scores and variance estimates for each group at all time points.
- Provide details on the sample characteristics and inclusion criteria for research with a healthy aging sample. If able, include information on age range, sex, socio-demographics, education, cognitive health, health status and related comorbidities, medications, different lifestyle aspects, and levels of functional independence.
- Conduct power analyses to adequately estimate the sample size and follow-up times needed to observe the effect of time on within-person changes in DTI parameters.

### 4.4 Conclusions and future directions

This study is the first attempt to synthesize observational longitudinal changes in the adult WM microstructure, providing estimates of effect sizes, direction regional variability, and modifiers in changes in DTI parameters. We also provided specific recommendations to ensure comparability and reproducibility in future longitudinal studies on WM. Therefore, our results should serve as a reference point regarding the expected effect and sample sizes in designing observational or randomized clinical trials, with DTI of the WM as the outcome variable. Furthermore, our results suggest that many protective and risk factors influence within-person deterioration in WM microstructure and, thus, may provide a good return on investment in clinical trials aimed at slowing down or reversing this decline using both lifestyle and pharmaceutical means. Our findings in healthy samples show promise of within-person DTI changes servicing as an early, preclinical indicator of risk of dementia, opening windows for early, more effective interventions and treatments.

Moving forward, several promising avenues of research can be explored:

1. Conducting a more detailed investigation of sex differences and possibly sex-specific modifiers of WM change.
2. Further investigation of the effects of aerobic exercise and cognitive training on WM changes is required through longitudinal studies with larger sample sizes and consistent follow-up times. Examining the combined effects of these modifiers and lifestyle interventions could help identify optimal strategies for promoting healthy aging WM.
3. Further investigation of the effects of diet, vascular risk factors (e.g., smoking), sleep, and alcohol consumption on WM changes and their interactions with biological sex.
4. Studying change in WM using advanced MRI methods, more sensitive to the WM’s microstructural tissue components and water-tissue interactions (Weiskopf et al., 2021), such as myelin-water imaging, advanced diffusion imaging, quantitative susceptibility mapping, or quantitative magnetization transfer. Despite its sensitivity to age-related differences and changes, DTI provides limited insight into the integrity of myelin and axons. Related to this, it must be noted that FA is a derivative of axial and radial diffusivities, and so is MD. Thus, the different metrics obtained by DTI are not mathematically independent, and their correlations may differ as a function of both local microstructure and age-related processes (Burzynska et al., 2010). Such findings highlight the challenge of identifying specific biological processes with DTI metrics. Longitudinal studies using more advanced techniques are currently emerging (Beck et al., 2021) and will help identify the biological underpinnings that drive changes in DTI metrics. This may facilitate the development of new non-pharmacological and pharmacological interventions targeting WM pathology to complement efforts focused on gray matter pathology in aging and dementia.

## Data Availability

This study primarily worked with summary effect sizes and individual-level data, which were obtained directly from the authors of the original studies or extracted from the original publications. In compliance with data sharing policies and respect for the confidentiality of individual-level data, we can provide a detailed summary of the effect sizes upon reasonable request.

## Appendices

**Appendix A.** Studies that contain longitudinal DTI data but do not report the effect of time on WM or do not have a healthy control group.

Authors	Year	Country/Study	Follow-up	N, % female	Age (y)	DTI measure	WM regions	Type of data/statistics reported	Main results	Moderators
Fletcher et al.	2013	USA/Longitudinal Cohort of the Alzheimer Disease Center at UC Davis	4.4 y converter to MCI3.8 y nonconverters	102, 67%20 converters82 nonconverters	M = 71.4	FA, RD, AD	FX	Regression and Cox proportional hazard rations for conversion to MCI, no statistics for longitudinal ΔDTI reported	Not reported	AD associated with conversion to MCI and dementia
Lampit et al.	2015	Australia/Timecourse Trial	3 and 12 weeks (two follow-ups)	Cog. Training: 7, 0%Active control: 5, 86%	M = 72M = 70	FA, MD, RD, AD	WM skeleton	Whole WM M±SD at t1, t2, t3, no statistics for longitudinal ΔDTI in controls reported	Not reported	No effect of the intervention on WM
Fissler et al	2017	Germany	10 weeks	Cog training:11, 55%Exercise: 12, 75%Control: 16, 50%	M = 71M = 74M = 71	FA	GCC, FX, CING hippocampus	β and 95% CI for the effect of time at post-test and 3-month follow-up in the control group	Not reported	No effects of the intervention or lifestyle physical activity on DTI metrics
Scott et al.	2017	USA/ADNI	6, 12, 24, and 36 months	HC: 46, 50%Early MCI: 48, 44%Late MCI: 29, 28%Dementia: 39, 36%	M = 72.9	FA, MD, RD	46 tracts grouped in component 1 (GCC, BCC, SCC, SS, ACR, SCR and PCR), component 2 (ALIC, PLIC, EC, SLF, CING), component 3 (CP, CST), component 4 (hippocampal WM)	LME models (MD component-by-time β as a predictor of cognitive change).Time effects not reported	Not reported	Late MCI and dementia: Δ MD predicted executive function↓ in components 1 and 2. Dementia: Δ MD predicted executive function↓ in component 4.
Racine et al.	2019	USA/Wisconsin Registry for Alzheimer’s Prevention. Patients at with family history of Alzheimer’s disease.	2 y	151, 68%	44-79	FA, MD	Whole WM, CING gyrus, CING hippocampus	β and 95% CI for the association of baseline biomarkers with ΔFA and MD, no statistics for longitudinal ΔDTI reported	Not reported	CSF biomarker YKL-40Δ: greater MD↑ in CING hippocampus
Staffaroni et al.	2019	USA/Hillblom Aging Network cohort	2.3 y	136, 54%	47-89	MD	Whole WM	Correlation of ΔMD with cognition or perfusion, no statistics for longitudinal ΔDTI reported	Not reported	↓ in global perfusion: greater ΔMD, processing speed↓
Maltais et al.	2020	France/ Multidomain Alzheimer’s PreventiveTrial (MAPT)	5 y, 3 t	167, 60%	M = 75	FA, MD	Regions on WM skeleton: CR, CC, EX, thalamic radiations, SLF, UN, CING hippocampus	Correlations of baseline physical activity with ΔFA and ΔMD, time effects not reported, no statistics for longitudinal ΔDTI reported	Not reported	Low active adults: greater MD↑ in UN
Raffin et al.	2021	France/ Multidomain Alzheimer’s PreventiveTrial (MAPT)	3 y	160, 63%	M = 75	FA, MD, RD, AD	Regions on WM skeleton: SLF, FX, SS, ALIC, ATR, STR, PTR, SCR	Correlations of baseline physical activity with ΔDTI by APOE genotype group, no statistics for longitudinal ΔDTI reported	Not reported	APOE e4 carriers: higher light physical activity (but not MVPA), greater MD↑ and AD↑ (controlled for age, sex, BMI, WMH, MAPT group, cardiovascular morbidities)

AD: axial diffusivity, ACR: anterior corona radiata, ALIC: anterior limb of internal capsule, ATR: anterior thalamic radiation, BCC: body corpus callosum, BMI: body mass index, BP: blood pressure, CC: corpus callosum, CING: cingulum, CP: cerebral peduncles, CST: corticospinal tract, EX: external capsule, FA: fractional anisotropy, FX: fornix, GCC: genu corpus callosum, HC: healthy controls, IC: internal capsule, IFOF: inferior frontal-occipital fasciculus, ILF: inferior longitudinal fasciculi, MCI: mild cognitive impairment, MD: mean diffusivity, PCR: posterior corona radiata, PLIC: posterior limb of internal capsule, RD: radial diffusivity, SCC: splenium corpus callosum, SCR: superior corona radiata, SLF: superior longitudinal fasciculus, SS: sagittal stratum, SFOF: superior fronto-occipital fasciculus, UN: uncinate, WM: White matter, WMH: white matter hyperintensities, YKL-40: Chitinase-3-like protein 1 (marker of glial activation and inflammation), Δ: change, m: month, y: year, t: timepoint.

**Appendix B.** NIH quality assessment tool for observational cohort studies.

Study	Clarity of objective	Specified population	Participation rate ≥ 50%	Uniform criteria	Sample size justification	Exposure before outcome	Sufficient timeframe	Consistent exposure measures	Multiple exposure assessments	Consistent outcome measures	Blinded assessors	Loss to follow-up ≤20%	Adjustment for confounders
Mielke et al. (2009)	-	-	-	-	-	-	+	-	-	-	-	-	-
Sullivan et al. (2010)	-	-	-	-	?	-	-	-	-	-	?	-	-
Barrick et al. (2010)	-	-	-	-	-	-	-	?	-	-	?	?	-
Charlton et al. (2010)	-	-	-	-	?	-	-	-	-	-	?	-	-
Teipel et al. (2010)	-	-	-	-	?	-	+	-	-	-	?	?	-
Pfefferbaum et al. (2014)	-	-	-	-	?	-	-		-	-	-	-	-
Sexton et al. (2014)	-	-	-	-	?	-	-	-	-	-	-	-	-
Lövdén et al. (2014)	-	-	-	-	?	-	-	-	-	-	?	+	-
Hakun et al. (2015)	-	-	-	-	?	-	-	-	-	-	-	?	-
Ritchie et al. (2015)	-	-	-	-	?	-	-	-	-	-	-	?	-
Vik et al. (2015)	-	-	-	-	?	-	-	-	-	-	-	-	-
Bender, Prindle, et al. (2016)	-	-	-	-	-	-	-	-	-	-	?	?	-
Bender, Völkle, et al. (2016)	-	-	-	-	-	-	-	-	-	-	?	?	-
Storsve et al. (2016)	-	-	-	-	?	-	-	-	-	-	?	-	-
Rieckman et al. (2016)	-	-	-	-	?	-	-	-	-	-	?	?	-
Köhncke et al. (2016)	-	-	-	-	?	-	-	-	-	-	-	-	-
Song et al. (2018)	-	-	-	-	-	-	-	-	-	-	-	-	-
Benitez et al. (2018)	-	-	-	-	-	-	-	-	-	-	-	?	-
Staffaroni (2018)	-	-	-	-	?	-	-	-	-	-	-	-	-
Williams (2018)	-	-	-	-	?	-	-	-	-	-	-	?	-
Kocevska et al. (2019)	-	-	-	-	-	-	-	-	-	-	-	-	-
Nicolas et al. (2020)	-	-	-	-		-	-	-	-	-	?	?	
Beck et al. (2021)	-	-	-	-	?	-	-	-	-	-	?	?	-
Coelho et al. (2021)	-	-	-	-	?	-	-	-	-	-	?	?	-

+ High risk of bias; - Low risk of bias; ? Risk of bias could not be assessed due to insufficient information.

**Appendix C.** NIH quality assessment tool for randomized controlled trials.

Study	Randomization	Concealed allocation	Blinding	Outcome assessor blinding	Baseline similarity	Low drop-out rate	Low differential drop-out rate	Adherence	Similar interventions	Valid outcome measures	Adequate sample size	Prespecified outcomes	Intention-to-treat analysis
Lövdén et al. (2010)	?	-	?	-	-	-	-	?	-	-	?	-	-
Engvig et al. (2012)	-	-	?	-	-	-	-	-	-	-	?	-	-
Voss et al. (2013)	-	-	-	-	-	-	-	-	-	-	-	-	?
Cao et al. (2016)	-	-	?	-	-	-	+	+	-	-	?	+	?
Burzynska et al. (2017)	-	-	-	-	-	-	-	-	-	-	-	-	-
De Lange et al. (2017)	-		?	-	-	-	-	-	-	-	?	-	-

+ High risk of bias; - Low risk of bias; ? Risk of bias could not be assessed due to insufficient information.
